# Prolonged magnesium sulfate infusion as adjuvant analgesia in postoperative transplant patients in the pediatric ICU: Preliminary results of a feasibility study

**DOI:** 10.1002/pne2.12131

**Published:** 2024-08-13

**Authors:** Joseph C. Resch, Shelby Graf, Ranad Ghalban, Srinath Chinnakotla, Gwenyth Fischer

**Affiliations:** ^1^ Department of Pediatrics, Division of Critical Care University of Minnesota Minneapolis Minnesota USA; ^2^ Department of Pediatrics, Division of Critical Care Michigan State University College of Human Medicine Grand Rapids Michigan USA; ^3^ University of Minnesota Medical School Minneapolis Minnesota USA; ^4^ Department of Surgery, Division of Transplantation University of Minnesota Minneapolis Minnesota USA

**Keywords:** analgesia, children, magnesium sulfate, PICU, prolonged infusion

## Abstract

The opioid crisis has emphasized identification of opioid‐sparing analgesics. This study was designed as a prospective trial with retrospective control group to determine feasibility for implementing a high‐dose prolonged magnesium sulfate infusion for adjuvant analgesia in the pediatric intensive care unit. Approval was granted for study of children receiving total pancreatectomy with islet cell autotransplantation and liver transplantation ages 3–18 years. Study exclusions were pregnancy, neuromuscular disease, hypersensitivity, preoperative creatinine >1.5 times upper limit normal, arrhythmia or pacemaker presence, and clinician concern. Eleven patients were enrolled between January 2020 and December 2022. Magnesium sulfate bolus (50 mg/kg) followed by intravenous infusion (15 mg/kg/h) was initiated in the operating room and extended postoperatively (maximum 48 h). Serum magnesium levels were monitored serially. To prioritize safety, infusion dose was decreased by 5 mg/kg/h for levels greater than 3.5 mg/dL. Clinical team otherwise followed standard multimodal pain practice. Primary outcome was oral morphine equivalent per kg per day during intensive care course (maximum 7 days). Secondary outcomes focused primarily on magnesium safety, including hemodynamic variables, electrolyte variables, respiratory support, and opioid‐related side effects. There were no serious adverse events. Treatment group trended toward slightly higher intravenous fluid requirement (~1 bolus), however no increase in blood product. Treatment and control groups were otherwise comparable in targeted outcomes and overall adverse event profile. Use of a high‐dose magnesium sulfate infusion protocol for analgesic postoperative use in select transplant recipients appears feasible for continued optimization of study in the PICU.

## 1 INTRODUCTION

Management of pain in the pediatric intensive care unit (PICU) requires a multimodal therapeutic approach to optimize risk–benefit profiles of complex clinical conditions. Postoperative populations inherently suffer acute pain, and protocols extending to children have been developed for enhanced recovery after surgery (ERAS), demonstrating improvement in clinical outcomes, satisfaction, and healthcare cost.^1^ Despite this, undertreated pain is associated with persistent decline of quality of life beyond the perioperative period.^2^ Opioids are a mainstay analgesic in critically ill patients, though the opioid epidemic remains prominent in the USA^3^ and is not limited to adults.^4^, ^5^, ^6^ Adolescents demonstrate persistent opioid use (beyond 3 months) in trauma^7^ and postsurgical populations,^8^ with resultant outpatient implications on later use.^9^ Amongst a myriad of side effects, opioids contribute to pediatric delirium,^10^ constipation,^11^ and risk of withdrawal,^12^ particularly at higher doses and duration. Parental stigma may alter opioid prescriptions.^13^ Drug shortages threaten inpatient availability of opioids and other analgesics.^14^, ^15^

Identifying opioid‐sparing and adjuvant analgesic therapies is therefore a national health focus. Magnesium sulfate (MgSO_4_) is a versatile agent which may act as an opioid‐sparing pain medication via its antagonistic effect on N‐methyl‐D‐aspartate (NMDA) receptors in the central nervous system.^16^ Initially trialed by Tramer et al, MgSO_4_ has been studied clinically in adults via a wide range of protocols.^17^, ^18^ Recently the analgesic property of MgSO_4_ has been investigated in pediatrics, demonstrating suggested benefit in operative populations.^19^, ^20^, ^21^, ^22^, ^23^ However, there has been mixed results depending on study design.^24^, ^25^, ^26^ Whether proven efficacious or not, these studies consistently reported no clinically meaningful adverse effects. Support for the safety of high‐dose prolonged infusions additionally exists in pediatric status asthmaticus protocols.^27^, ^28^

Postoperative transplant recipients offer a critically ill population which ubiquitously requires high opioid use, often requires prolonged admissions in the PICU, and carries a hemodynamic profile that may challenge the side effect profile that limits MgSO_4_ use, therefore lending an opportune initial population to trial use of adjuvant therapies for optimizing pain regimens. To our knowledge, transplanted children have not been studied with MgSO_4_ for analgesia. We hypothesized that the addition of a novel high‐dose prolonged IV (intravenous) MgSO4 infusion protocol to preestablished analgesic regimens in transplant recipients can decrease IV morphine equivalent usage in the pediatric ICU. The purpose of this study was to pilot a MgSO_4_ protocol at our institution to establish safe and reliable delivery, help guide dosing optimization, and contribute to expanding literature.

## 2 MATERIALS AND METHODS

Extensive literature review was performed to identify available physiologic data, appropriate dosing options, and side effect profile of MgSO_4_. Assistance from the clinical research support center (CRSC) at the University of Minnesota (UMN) was incorporated for feasibility review and study development. Critically ill patient populations expected to have high opioid requirements were considered. There was synchronous focus on opioid‐sparing therapy with our institution's pediatric transplant surgeon, so postoperative solid organ transplant patients were selected. Both transplant populations (TPIAT, LT) have clinical protocols which include a comprehensive treatment pathway to standardize decisions regarding hemodynamics (vital parameters, fluids, vasopressors), nutrition, and pain; the latter of which includes use of our institution's pediatric pain/palliative specialist. Ketamine and dexmedetomidine are two adjuvant therapies with expected variance within these protocols. Given that MgSO_4_ is not approved for analgesia and pediatric transplant recipients have not been studied for this purpose, this protocol required approval as an investigational drug (IND) via the U.S. Food and Drug Administration (FDA). Approval was additionally granted by the UMN institutional review board for initiation in January 2020, and enrollment on clinicaltrials.gov (NCT04812028) occurred prior to patient recruitment or data collection. Ethics of data integrity, consent and assent were upheld. Due to COVID‐19 regulations study enrollment did not begin until July 2020.

Power analysis was performed due to limited available analgesic consumption data in pediatric liver transplant (LT) and total pancreatectomy and islet cell autotransplantation (TPIAT) recipients.^21^, ^22^, ^29^, ^30^ Patient target was 30, and grant funding was secured for an anticipated 3 year enrollment. Inclusion criteria for consideration of enrollment were: age 3–18 years at screening, informed consent by patient's legal representative (additional assent if age‐appropriate), scheduled operation of either LT or TPIAT; exclusion criteria included: pregnant female, presence of a condition or abnormality that in the opinion of the investigator would compromise patient safety, allergy to components of MgSO_4_, history of heart block or myasthenia gravis, presence of cardiac pacemaker, preoperative creatinine level >1.5 times the upper limit of normal, renal transplant recipient. There was no exclusion based on race, ethnicity, social status, or sexual identity. Patients younger than 3 years were excluded in the IND based on potential concerns of effect on neurodevelopment in animal models.^31^ A retrospective observational control group meeting the same inclusion/exclusion criteria was to be analyzed in a 2:1 ratio to help determine comparative trends.

Patients who satisfied study criteria were approached for consent. Multidisciplinary providers were notified of an enrolled patient (transplant surgery, anesthesiology, PICU). Participants received IV MgSO_4_ bolus 50 mg/kg [max 2 g] over 30 min in the operating room (OR) immediately following anesthetic induction (in TPIAT arm) or following liver reperfusion (in LT arm). This was followed by initiation of an IV MgSO_4_ infusion 15 mg/kg/h (max 2 g/h) for up to 48 h extending into their PICU admission. Anesthesiologists were encouraged to treat hemodynamics per their standard of care. Serum magnesium levels were monitored every 2 h in the OR, then every 4 h in the PICU throughout infusion duration. The infusion dose was decreased by 5 mg/kg/h for any serum level >3.5 mg/dL. To our knowledge there is no known or established therapeutic serum magnesium level in adults or pediatrics for analgesia, only infusion dosages which are suggested to benefit, so there were otherwise no targeted titrations. If infusion dose decreased to 0 mg/kg/h per protocol (i.e. if three levels >3.5 mg/dL), this was not considered early discontinuation, but rather appropriate titration per protocol. This approach was designed alongside discussions with the FDA to prioritize safety, given expectations to frequently encounter hemodynamic insufficiency in the selected populations independent of MgSO_4_ therapy. Discretion was allowed for providers to temporarily pause infusion if considering magnesium as a potential cause of clinical side effect, with intention that it is restarted if deemed unrelated to study drug. Once enrolled, patient data was collected on an intent‐to‐treat basis, regardless of when infusion was stopped. Study drug was stored and distributed by the investigation drug service (IDS) pharmacy at the University of Minnesota Medical Center. Anesthesiologists and critical care physicians were instructed to otherwise treat patient pain as they would per standard transplant approaches.

Data collected from patient electronic medical records were transferred to source documents and stored in REDCap clinical database for 4 patient arms: TPIAT treatment (TP‐Mg), TPIAT control (TP‐Co), LT treatment (LT‐Mg), LT control (LT‐Co). The primary outcome was oral morphine equivalents (OME) per kg per day while in the PICU, with additional exploration according to postoperative day (POD) of treatment. Calculation of OME was according to methodology of Nielsen et al with attention to United States recommendations when present.^32^ Secondary outcomes focused on opioid side effect data, pain score trends, PICU outcomes, and magnesium data (serum levels, side effects, dosing). Hemodynamic evaluation included vasoactive‐inotropic scores (VIS) as a quantifiable surrogate value for vasopressor load, calculated using formulas originally proposed by Wernovsky et al,^33^ recognizing this is not validated specifically for our populations. Data elements are provided in supplementary figure S1. Data were collected for day of operation (including OR course) and for 7 postoperative days (7 am – 7 am) or transfer from PICU, whichever was first. Intention was for uni‐ and multivariate biostatistical analysis to identify statistical significance (*p* < 0.05), however ultimately given the size of the enrolled prospective cohort, statistics were focused on observational findings only. Recommendations of final statistical observations were provided through biostatistician support through the clinical and translational science institute (CTSI) at UMN.

A data safety monitoring board (DSMB) evaluated safety data serially with enrollment. Additional safeguard monitoring occurred through standard reports and auditing via the FDA, IRB, and CTSI at UMN. Adverse event reporting followed IRB and FDA standard safety protocols per CTCAE (common terminology criteria for adverse events).

## 3 RESULTS

**FIGURE 1:**
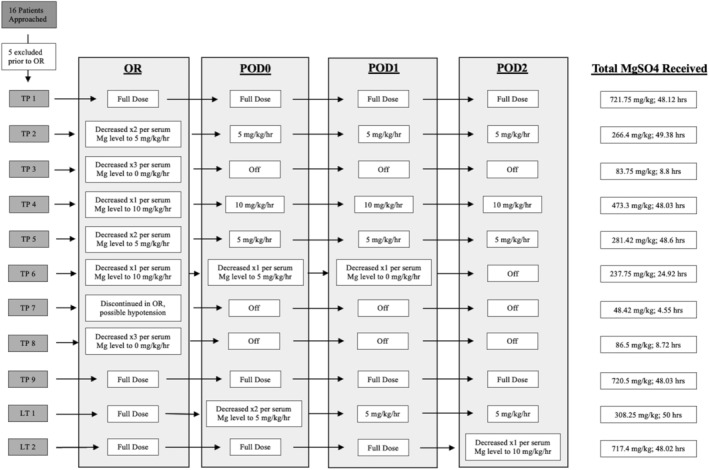
Patient flow summary. Enrollment numbers and infusion dosing changes by patient in operating room and PICU postoperative Days 0–2. LT, liver transplantation; Mg, magnesium; MgSO_4_, magnesium sulfate; OR, operating room; POD, postoperative day; TP, total pancreatectomy & islet cell autotransplantion.

Demographic data and PICU length of stay is provided in Table 1. Populations were evaluated independently given surgical and inherent clinical differences. Sixteen patients were approached and 11 consented for study (9 TPIAT, 2 LT). Treatment arm had a median age of 16 years [IQR 4], median weight of 61.4 kg [IQR 17.35], and median PICU length of stay of 5.8 days [IQR 3]. Enrollment was lower than predicted primarily due to institutional effects of the COVID pandemic. Retrospective control data was collected in parallel during the study and reached higher than the required 2:1 ratio, all of which were included in analysis (34 TP‐Co, 21 LT‐Co), allowing better representation of comparative data. A patient flow summary is provided in Figure 1, which outlines enrollment and dosing changes by postoperative day. Magnesium sulfate was turned off twice for hypotensive evaluation (once PICU, once OR), neither of which had a determined MgSO_4_ causality, though was left discontinued prophylactically in the OR case. The highest recorded Mg level was 3.8 mg/dL in LT‐Mg and 4.2 mg/dL in TP‐Mg. Two patients (18%) maintained the full infusion dose for 48 h. Four patients (36%) were discontinued prior to 48 h and 3 of those prior to reaching the PICU. There were 2 protocol deviations, both of which involved early spacing or discontinuation of magnesium levels by the clinical team, resulting in modifications to improve communication and increase direct study team presence. There were no serious adverse events reported throughout.

**TABLE 1:** Demographics, length of stay, and respiratory support needs.

	Age (years)		Sex (%)		Weight (kg)		PICU Days	
	Mean (SD)	Median [IQR]	Male	Female	Mean (SD)	Median [IQR]	Mean (SD)	Median [IQR]
TPIAT								
MgSO_4_ (*n* = 9)	14.8 (2.17)	16 [4]	0.33	0.67	59.9 (11.6)	61.4 [13.8]	7.38 (2.92)	6.74 [1.98]
Control (*n* = 34)	12.3 (4.23)	14 [6.75]	0.38	0.62	47.6 (18.2)	49.5 [28.6]	7.29 (2.67)	6.74 [2.32]
LT								
MgSO4 (*n* = 2)	11 (7.07)	11 [5]	0	1	41.6 (34.79)	41.6 [24.6]	4.19 (0.42)	4.19 [0.3]
Control (*n* = 21)	8.71 (4.33)	8 [7]	0.57	0.43	36.3 (24.22)	23.6 [34.8]	8.47 (6.97)	5.94 [5.77]
TPIAT + LT								
MgSO_4_ (*n* = 11)	14.1 (3.33)	16 [4]	0.27	0.73	56.6 (16.8)	61.4 [17.35]	6.8 (2.92)	5.8 [3]
Control (*n* = 55)	11 (4.56)	12 [8]	0.47	0.53	43.44 (21.2)	43.7 [36.6]	7.75 (4.76)	6.68 [3.27]

Abbreviations: IQR, interquartile range; LT, liver transplant; MgSO_4_, magnesium sulfate; PICU, pediatric intensive care unit; SD, standard deviation; TPIAT, total pancreatectomy & islet cell autotransplantation.

**FIGURE 2:**
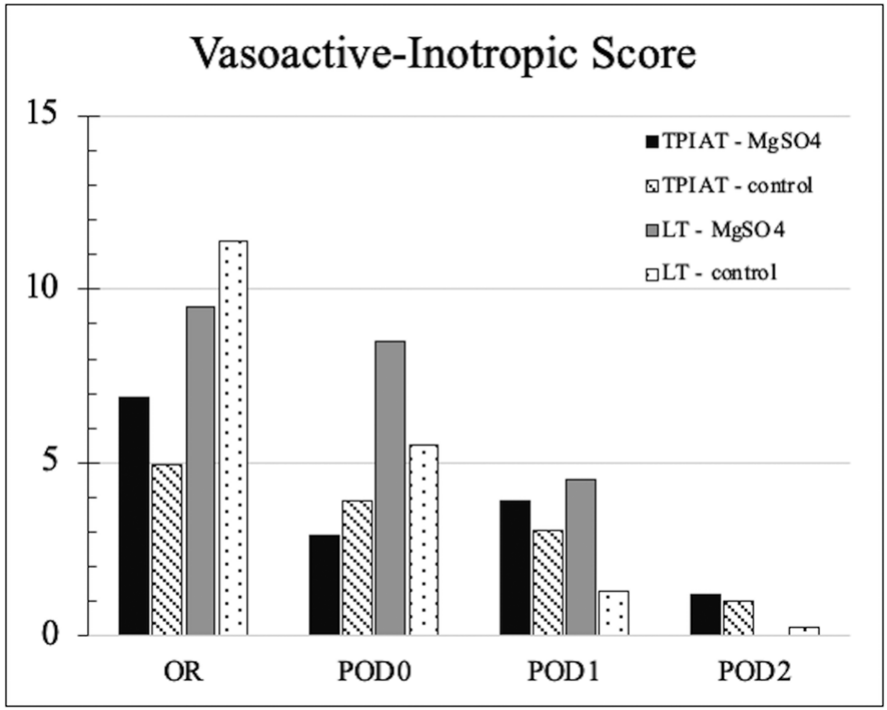
Vasoactive‐inotropic scores. Average peak vasopressor infusion dose displayed in operating room and PICU postoperative Days 0–2. LT, liver transplantation; Mg, magnesium; POD, postoperative day; TP, total pancreatectomy & islet cell autotransplantation.

**FIGURE 3:**
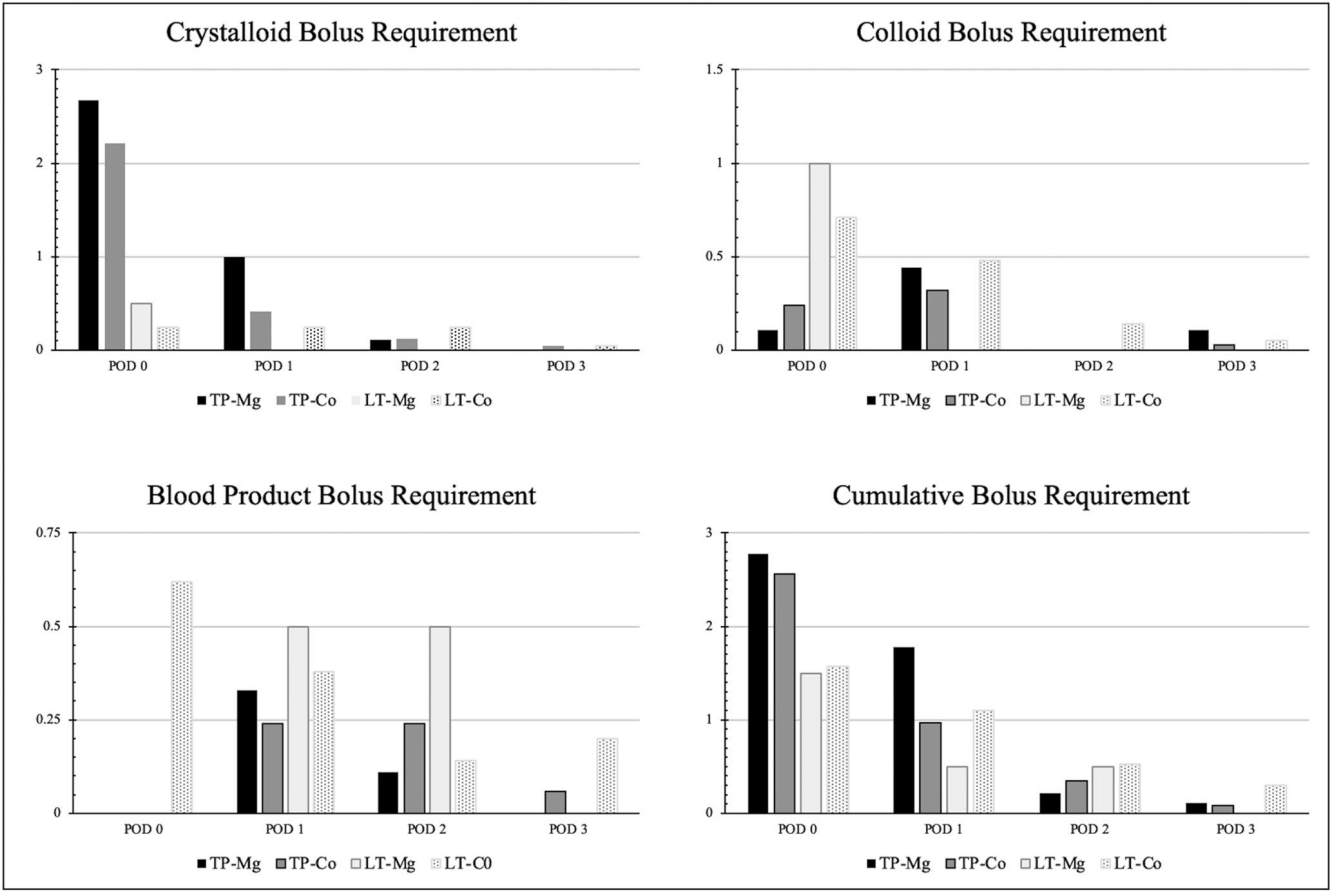
Intravenous fluid boluses. Average intravenous fluid bolus requirements for PICU postoperative Days 0–3 during or proximal to magnesium sulfate infusion. Co, control; LT, liver transplantation; Mg, magnesium; POD, postoperative day; TP, total pancreatectomy & islet cell autotransplantation.

Hypotension was present in 81% of patients in treatment arm and 80% in control arm, with one patient in treatment arm having hypotension that led to early discontinuation of MgSO_4_, which on review was deemed ‘possibly’ associated. Data were evaluated to capture meaningful interventions for blood pressure or hemodynamic change. Average peak pressor medication is depicted in Figure 2. Patients in LT‐Mg on POD1 and POD2 had the largest apparent difference but remained low overall (low‐moderate pressor requirement in LT‐Mg and LT‐Co on POD1, low pressor requirement in LT‐Mg and LT‐Co on POD2). Supplementary table S1 outlines OR interventions, which anecdotally included a wide range of anesthetic approach depending on provider. Despite having less sustained hypotensive events, there was apparent increased use of phenylephrine and ephedrine in the MgSO_4_ groups, the two most common hypotensive rescue medications. Sporadic use of norepinephrine (1) and vasopressin (4) boluses were also used in LT‐Co. There was otherwise no increase in fluid, blood, or calcium requirement in MgSO_4_ groups, and relevant electrolytes were in similarly normal ranges. Postoperative IV fluid bolus requirements most proximal in time to the MgSO_4_ infusion (POD 0–3) are shown in Figure 3. There was an increase in TP‐Mg fluid interventions on POD1 and LT‐Mg blood product requirement on POD2, though each by <1 bolus difference from control. Summative blood product bolus requirement for POD 0–3 was less in both TP‐Mg (0.44 vs. 0.54) and LT‐Mg (1 vs. 1.34). Summative volume requirement for POD 0–3 was ~1 bolus higher in TP‐Mg (4.89 vs. 3.97) primarily due to crystalloid use on POD0 and POD1.

Magnesium effects on pertinent electrolytes and renal function are demonstrated in supplementary table 2. Magnesium peak and daily average levels were the only notable clinical difference, though the TP‐Mg magnesium levels were near normal by POD1. Common electrolyte repletion requirements (potassium, phosphorus, magnesium) appear decreased in MgSO_4_ groups. There were no hyperkalemic events requiring treatment. Hypomagnesemia was prevalent in LT groups (0.67% LT‐Co, 100% LT‐Mg) and TP‐Mg posttreatment (0.33% vs. 0.12% TP‐Co), though control groups had overall infrequent serum Mg surveillance. Average creatinine levels were comparable. One patient in LT‐Mg (vs. 4 in control) had a Cr >1.5x upper limit of normal, occurring after the infusion period. No patients in treatment groups required dialysis (2 in LT‐Co).

Targeted adverse event prevalence and commentary is outlined in Table 2. Most events were comparable between groups, and none were definitively deemed secondary to MgSO_4_ infusion in DSMB or research team review. Notably, pulmonary edema or effusion was found in two TP‐Mg patients, with one of these receiving increased IV fluid boluses compared to average control in both OR and early PICU (identified retrospectively once cumulative data finalized). This patient had additional technical aspects of surgery which were deemed contributory to the hemodynamic profile, including portal venous compression and significant collateral vasculature. Respiratory depression is an overlapping side effect of both MgSO_4_ and opioids, and mechanical ventilation dependence may drive increased opioid need, so was further evaluated. The average POD at extubation for TPIAT arm was 0.11 (SD 0.33) in MgSO_4_ cohort and 0.18 (SD 0.46) in controls; for LT arm was 0 (SD 0) in MgSO_4_ cohort and 2.21 (SD 2.18) in controls. This included excluding 2 LT patients due to extubation beyond the recording period. The percentage of days requiring either NIPPV or IMV in the TPIAT arm averaged 0.17% (SD 0.16) for MgSO_4_ cohort and 0.14% (SD 0.09) in controls; for LT arm averaged 0.4% (SD 0.57) for MgSO_4_ cohort and 0.57% (SD 0.31) in controls. Delirium was higher in TP‐Mg but was not attributed to study drug in all cases. Other adverse events encountered but not related to magnesium included chylous ascites (1), gastroparesis (2), abdominal bleeding (3), lactic acidosis (1), hemorrhoids (1), GJ displacement (1), ropivacaine toxicity (1), portal hypertension (1). There were no evident effects on physical therapy initiation or progress.

**TABLE 2:** Adverse event prevalence (%). Hypotension was considered if BP was lower than surgeon/team goals. Bradycardia if <5th %tile for age. Prolonged hypoxia if persistent low‐flow supplemental oxygen requirement beyond expectation per progress note. AKI if creatinine elevated for age at this institution. Infection if positive culture (not necessarily clinical).

	TPIAT		LT		Total		
	MgSO_4_ (*n* = 9)	Control (*n* = 34)	MgSO_4_ (*n* = 2)	Control (*n* = 21)	MgSO_4_ (*n* = 11)	Control (*n* = 55)	Pertinent MgSO_4_ comments
Hypotension	0.78	0.79	1	0.81	0.81	0.8	
Bradycardia	0.33	0.24	0	0.62	0.27	0.38	Attributed to dexmedetomidine in 1, others either already off MgSO_4_ or levels already normalized
Arrhythmia (other)	0.11	0.06	0.5	0.05	0.18	0.05	Sinus pauses—resolved w/ dexmedetomidine off; QTc (494 ms)—resolved spontaneously
Delirium and/or hallucinations	0.56	0.21	0	0.19	0.45	0.2	Occurred in 4 pts while MgSO_4_ infusion off, 1 pt due to ketamine; no pts w/formal delirium scoring
Pulmonary Edema or Effusion	0.22	0.06	0.5	0.33	0.27	0.16	Edema deemed unlikely due to MgSO_4_, however review noted increased IVF blousing in second TPIAT case, and infusion was present in only LT case
Infection	0.44	0.44	0	0.19	0.36	0.35	Primarily intraoperative culture positivity in both groups
Prolonged Hypoxia (other)	0.11	0.03	0	0.05	0.09	0.04	Attributed to persistent atelectasis
Anaphylaxis	0	0.03	0	0	0	0.02	
Abnormal movement or sensation	0	0.06	0.5	0	0.09	0.04	Abnormal mm jerking and tongue protrusion, deemed unrelated
AKI	0	0.03	0.5	0.33	0.09	0.15	Routine Cr check, MgSO_4_ already off >24 h
Flushing	0	0	0	0.19	0	0.07	
Rash (other)	0	0.03	0	0.05	0	0.04	
Dizziness	0	0.06	0	0	0	0.04	

Abbreviations: AKI, acute kidney injury; IVF, intravenous fluid; LT, liver transplantation; Pt, patient; TPIAT, total pancreatectomy & islet autotransplantation.

**FIGURE 4:**
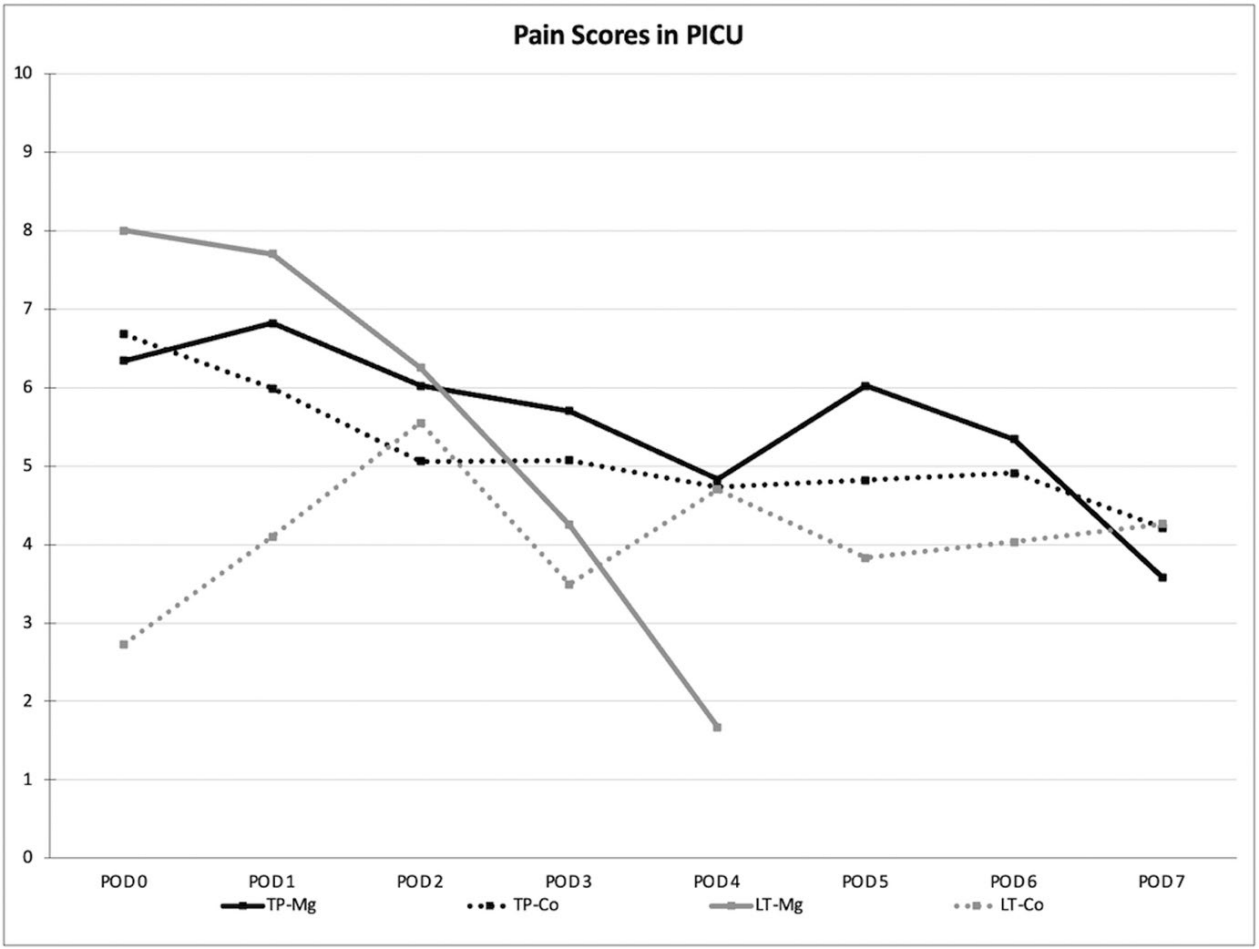
Pain Scores. Average verbal pain score by postoperative day in PICU. Co, control; LT, liver transplantation; Mg, magnesium; POD, postoperative day; TP, total pancreatectomy & islet cell autotransplantation.

Data regarding opioid consumption and related side effects are provided in Table 3. There was no identifiable trend toward decreased opioids in TP‐Mg arm, however the infusion was decreased to 0 mg/kg/h prior to PICU arrival in 33%. Paravertebral block use was present in all TPIAT patients. Prevalence of any ketamine use varied (44.4% TP‐Mg; 67.6% TP‐Co; 0% LT‐Mg; 28.6% LT‐Co). Postoperative ketamine analgesic dose was higher in TP‐Co (average total dose 3.26 vs. 1.27 mg/kg in Tp‐Mg) as well as increased paravertebral block interventions (2.56 vs. 1.56 TP‐Mg). Postoperative dexmedetomidine use was utilized in all Mg patients, 97% TP‐Co, and 90% of LT‐Co; higher total dose was utilized in TP‐Mg (22.1 mcg/kg) then TP‐Co (13.7 mcg/kg). There was earlier extubation in the LT‐Mg group (100% extubated on POD0 in LT‐Mg vs. 24% in LT‐Co). When controlling for this difference, opioid use remained decreased for both 48‐h (4.3 mg/kg/d vs. 5.68 in LT‐Co) and total dose (3.31 mg/kg/d vs. 6.39 in LT‐Co) in the LT‐Mg group. Opioid‐related side effects were generally similar between groups, aside from TP‐Mg experiencing more inferences of delirium (as above) and on average ~1 additional emesis per admission. Pain score trends by postoperative day are depicted in Figure 4.

**TABLE 3:** Opioid data. Values presented as mean (standard deviation) for opioid consumption section, mean or prevalence (%) for adverse events section; pertinent commentary provided.

	*TPIAT*			*LT*		
	MgSO_4_ (*n* = 9)	Control (*n* = 34)	Comments	MgSO_4_ (*n* = 2)	Control (*n* = 21)	Comments
OME/kg						
Total PICU Course	5.27 (2.94)	4.1 (2.41)	Higher Ketamine use in control, Higher Dexmedetomidine use in MgSO_4_	3.31 (1.69)	10.2 (8.32)	Both MgSO_4_ pts extubated on POD0, confounding overall opioid requirement
POD0—POD2	5.33 (3.03)	4.97 (3.28)		4.3 (1.28)	14.06 (11.6)	
POD0	1.91 (1.23)	2.26 (2.21)	67% on MgSO_4_ infusion	2.12 (1.99)	7.02 (8.17)	MgSO_4_ infusion remained on through POD2 in both
POD1	5.39 (3.18)	5.37 (3.76)	67% on MgSO_4_ infusion	2.08 (2.3)	13.98 (11.58)	
POD2	5.74 (3.63)	4.76 (3.76)	56% on MgSO_4_ infusion	3.75 (1.96)	13.49 (13.2)	
OR	3.6 (2.51)	3.24 (1.44)	High variability in anesthetic & analgesic approach	2.19 (0.3)	4.69 (1.94)	High variability in anesthetic & analgesic approach
Adverse events						
Delirium or AMS (%)	0.56	0.21	Prevalence as indicated in progress notes	0	0.24	
Ileus (%)	0.56	0.62	Prevalence as indicated in progress notes	0	0.24	
Suppository or Enema use (%)	0.22	0.32	Prevalence of any use	0	0.24	
First Stool (POD#)	4	3.76		2	3.83*	*1 pt (4.7%) did not stool in control
Feed Initiation (POD#)	2.22	2.06		2	2.71	
Feed Goal Reached (POD#)	4.38*	4.53*	*2 pts (6%) did not reach goal in control, 1 pt (11%) did not reach in MgSO_4_, not reflected in calculation	4.5	5.1*	*11 pts (52%) did not reach goal feeds, not reflected in calculation
Emeses (total)	2.44	1.26	Total # emeses for PICU course	0	1.38	
Ondansetron (total doses)	11.44	10.79	Additional 6 pts received granisetron in control, 1 pt received ondansetron infusion in control	1	2.86	
Ondansetron (% any use)	0.89	0.94		0.5	0.43	
Urinary Retention (%)	0.22	0.18		0	0.1	
Naloxone Infusion (%)	0.78	0.59	Surrogate intervention for pruritus; any dose	0	0.14	

Abbreviations: AMS, altered mental status; LT, Liver transplantation; MgSO_4_, magnesium sulfate; OME, oral morphine equivalent; POD, postoperative day; TPIAT, total pancreatectomy & islet cell autotransplantation.

## 4 DISCUSSION

Given the physiologic plausibility via antagonism of NMDA receptors and successful use in both adult and pediatric populations, we used existing literature to guide development of a novel protocol using prolonged high‐dose MgSO_4_ infusion to begin a line of study determining its potential efficacy as an opioid‐sparing agent in the pediatric ICU.^18^, ^21^, ^22^ Magnesium sulfate's potential for hemodynamic consequence (hypotension, arrhythmias) lends hesitancy for use in critically ill populations, who often require hemodynamic support for other clinical problems, despite otherwise having a large supratherapeutic window before this is encountered.^16^ To be able to begin inferring applicability of MgSO_4_ for adjuvant use in the PICU, we therefore aimed to select populations reflective of some of these challenges, as well as expectations of high opioid need and potential for benefit. We chose two transplant populations (Liver and TPIAT) which met this profile, had coexisting interest in opioid‐sparing therapy from the institution's surgeon, and had preexisting standardized protocols for pain treatment, typically requiring opioid infusions for significant postoperative pain.

Our preliminary data suggests feasibility of implementing a MgSO_4_ infusion protocol for continued study of analgesia in a critically ill pediatric population. Most notably, there were no severe adverse events. There was a comparable hemodynamic profile to controls. Specifically, vasopressor use was in comparably low or moderate dosages for both groups in the early postoperative period, and cumulative intravenous fluid bolus requirement was similar (<1 bolus difference between treatment and controls). This included no increase in postoperative blood product requirement. Electrolyte profile was similarly comparable, with no escalated calcium repletion or need for hyperkalemic therapy. There was no trend toward escalated invasive or non‐invasive positive pressure requirement in treatment groups. Enrollment rate was 69%, with consent times of ~30 min. The implementation of the protocol requires attentive multi‐disciplinary communication to avoid protocol deviations, which were observed in two patients – one owing to early spacing of lab checks (transitioned to the standard clinical transplant protocol despite MgSO_4_ presence), and one owing to missed OR serum checks, both which resulted in minor protocol modifications and no effect on clinical course.

There should be cautious interpretation of any inferences regarding analgesic effects, and the lack of ability to reach enrollment target prevents any evaluation of true efficacy. The LT‐Mg group appeared to have a trend toward lower opioid requirements, even when controlled for intubation, however included only two patients. The TP‐Mg group conversely did not show trends toward efficacy, however also received more ketamine adjuvant compared to control, and did not account for effects of chronic opioid use (characteristic of these patients). Dexmedetomidine use was ubiquitous in all groups (at least 90% of patients in each arm received drug) and likely lends further confounding to interpretation of any efficacy observations given adjuvant analgesic properties. Future studies ideally should standardize dosing across populations to control for this effect, though encourage multimodal analgesia that can tailor to individual patient needs.

There were several limitations. Most notably, this was a single‐site study and enrollment was significantly lower than expected, owing to operational shutdowns with the COVID pandemic and difficulty with timely recruitment and consent of acute liver transplants. Additionally, the age restriction (exclusion for <3 years) decided on during FDA safety discussions prevented a significant percentage of LT opportunity. Decreased enrollment prevented any analysis for statistical significance, and therefore this study was ultimately not powered to observe trends in opioid use as initially intended, so the primary goals shifted to feasibility and viability of protocol to guide future research. Anesthesia practice was variable, which resulted in challenges comparing OR variables, as well as interpreting any effect operative regimens contributed to postoperative opioid consumption. Control population was primarily retrospective, and lack of blinding of medical providers or patient subjects may have induced detection bias and nocebo effects. Opioid dosing in electronic medical record for select cases included infrequent pump clearance, making it difficult to discern specific variance by POD (though total dosage was accurate). Patients in TP‐Co arm demonstrated more heterogeneity regarding analgesic practice than expected, including intermittent use of ketamine as primary pain option (vs. rescue or adjuvant therapy), which was typically due to patient‐specific opioid side effect profile identified prior to admission. Effect of open‐abdomen status at PICU arrival and repeat OR presentations in LT‐Co was difficult to interpret so not commented on but may have affected adverse event and opioid data. Pain intensity scores did not discern passive versus active, which may be advised.^34^ Pain scores/indicators and select opioid side effects may be both difficult to interpret as well as lack clinical meaning in critically ill populations.^35^, ^36^ There exists a lack of agreement for optimal design of pediatric pain studies^37^, ^38^ and our variables were not congruent with recent recommendations of the PROMT/IMI‐PainCare meeting^39^ including functional measures or long‐term effects, both which may be impactful but were beyond the scope of this project. Opioid equivalent calculations vary in the literature. Our source was chosen based on its intended applicability to research studies, reference within CDC guidelines, and inclusion of a wide range of opioids.^32^ The limitations of this feasibility study will inform the design of a larger MgSO_4_ trial.

Based on lessons learned from the study team, we recommend continued modification of the dosing protocol to optimize ability to truly determine whether MgSO_4_ would be a beneficial opioid‐sparing adjuvant in the PICU. Consideration of other optimal populations subject to benefit, as well as multi‐site collaboration for recruitment is paramount. Evaluation of operative use may be avoided if unable to uniformize anesthetic practice. Multidisciplinary collaboration with anesthesiology to develop a standardized sedation regimen to help control for both operative and postoperative effects on pain is otherwise recommended in trials powered for efficacy. Inability to maintain supratherapeutic Mg levels and optimal infusion dose may hinder ability to determine true efficacious potential of treatment. Our current dosing frequently reached levels >3.5 mg/dL, necessitating a decrease in dose per protocol, despite being consistently within a level which was similar or less than MgSO_4_ infusion use in other critical populations.^12^ An increase in this serum threshold may be advantageous. Additionally, high‐dose infusion use in asthma suggests safety with less serum checks than our current study (every 6 h), which would minimize line interrogation, iatrogenic blood loss, and cost, and be more in line with current standard of practice. Younger populations may also be subject to study if MgSO_4_ is not deemed to have negative impacts on neurological development. Data collection was lengthy (minimum ~2–3 h per patient) and included most generalized variables encountered in acute pain studies, but may be prudent to condense or use automated collection processes in larger studies.

## 5 CONCLUSION

In conclusion, use of a high‐dose prolonged infusion of magnesium sulfate appears feasible for continued study of opioid‐sparing potential in postoperative critically ill pediatric patients, specifically total pancreatectomy with islet cell autotransplantation and liver transplant recipients. There were no serious adverse events encountered with a preestablished serum magnesium threshold. No conclusions regarding analgesic efficacy of magnesium sulfate can be drawn from this feasibility study. Optimization of dosing and increased enrollment should be undertaken to better define safety and efficacy profiles. Further work is needed to determine applicability to pediatric critically ill patients.

## AUTHOR CONTRIBUTIONS

Joseph Resch: conceptualization, data curation, formal analysis, funding acquisition, investigation, methodology, project administration, visualization, writing‐original draft, writing‐review & editing. Gwenyth Fischer: conceptualization, investigation, project administration, supervision, writing‐review & editing (primary editor). Srinath Chinnakotla: conceptualization, supervision, writing‐review&editing. Shelby Graf: data curation, investigation, writing‐review & editing. Ranad Ghalban: data curation, investigation, writing‐review & editing.

## FUNDING INFORMATION

An internal grant awarded to the study team by the University of Minnesota Department of Pediatrics provided funding for this trial.

## CONFLICT OF INTEREST STATEMENT

The authors declare that the research was conducted in the absence of any commercial or financial relationships that could be construed as a potential conflict of interest.

## Supporting information


**Figure S1.** List of Data Variables. FDA, Food & Drug Administration; FLACC, face, legs, activity, cry, consolability score; Mg, magnesium; MgSO_4_, magnesium sulfate; OR, operating room; PCA, patient‐controlled analgesia; PICU, pediatric intensive care unit; PO, per oral (by mouth); PRN, pro re nata (as needed); TPIAT, total pancreatectomy & islet cell autotransplantation; VAS, visual analogue scale.

Data S1:

## Data Availability

The authors generated an original dataset for this study which is stored in University of Minnesota programming (REDCap and Box Storage). These data can be shared on request via contact with the corresponding author, who can help facilitate a data transfer request.
