# Supplementary material for: Prolonged magnesium sulfate infusion as adjuvant analgesia in postoperative transplant patients in the pediatric ICU: Preliminary results of a feasibility study
Source: Paediatr Neonatal Pain. 2024 Aug 13;6(4):203–12. doi: 10.1002/pne2.12131 (PMC11645970; doi:10.1002/pne2.12131)
Supplement: Supplementary file 1 — Figure S1. List of Data Variables. FDA, Food & Drug Administration; FLACC, face, legs, activity, cry, consolability score; Mg, magnesium; MgSO4, magnesium sulfate; OR, operating room; PCA, patient‐controlled analgesia; PICU, pediatric intensive care unit; PO, per oral (by mouth); PRN, pro re nata (as needed); TPIAT, total pancreatectomy & islet cell autotransplantation; VAS, visual analogue scale. [file PNE2-6-203-s002.pdf]

Appendix 1. List of Study Variables

Demographics
Gender
Age, Weight, Height
Date of Surgery
Transplant received (TPIAT/Liver)
Abdomen Left Open after Surgery (y/n)
PICU Length of Stay

OR Data
Ionized Calcium, Potassium Levels (average)
Total Fluid [non-blood] Required (mL)
Total Blood Product Required (mL)
Number of Pressor Infusions Required, Peak Pressor Doses
Vasoactive-Inotropic Score
Ephedrine, Phenylephrine, Vasopressin, Epinephrine, and Norepinephrine Bolus Rescue Doses
Bradycardia Present (yes/no), Estimated Time Bradycardic (minutes, % total)
Sustained Hypotensive events
Total OR time (minutes)
Calcium Chloride, Potassium Chloride Boluses (# received)

Opioid/Sedation Consumption and Side Effect Data
Opioid Class (by day and overall)
PO Morphine Equivalent/kg (by day, for postoperative day 0 – 2, and overall for discharge or max 7-day)
% opioid scheduled vs PRN/demand
PCA use (yes/no), PCA deliveries vs demands
Ketamine Dosage (mg/kg by day and overall for discharge or max 7-day), Ketamine Infusion Use (yes/no)
Dexmedetomidine Peak dose (mg/kg/hr, by day), Dexmedetomidine Total Dose (mg/kg, overall for discharge or max 7-day)
Paravertebral Block Use (yes/no), Number of paravertebral Block Interventions (considered increase in infusion dose or boluses)
Number of Stools (by day and overall for discharge or max 7-day), Day of 1st Stool (Postoperative day #)
Suppository or Enemas used (by day and overall for discharge or max 7-day)
Ileus Documented (yes/no)
Initiation of Enteral Feeds (postoperative day #), Reached Goal Feeds (postoperative day # or did-not-reach)
Emeses (by day and overall for discharge or max 7-day)
Ondansetron or Granisetron doses, Prochlorperazine or Metoclopramide doses (by day and overall for discharge or max 7-day)
Scopalamine patch use (yes/no, by day and overall for discharge or max 7-day)
Cyproheptadine doses, Diphenhydramine doses (by day and overall for discharge or max 7-day)
Benzodiazepine doses (by day and overall for discharge or max 7-day), Midazolam Infusion use (yes/no)
Acetaminophen doses (by day and overall for discharge or max 7-day)
Ketorolac doses, Ibuprofen doses, Celecoxib doses (by day and overall for discharge or max 7-day)
Gabapentinoid doses (by day and overall for discharge or max 7-day)
Tricyclic antidepressant doses (by day and overall for discharge or max 7-day)
Naloxone used (yes/no)
Urinary Retention (yes/no)
Delirium (yes/no per progress note documentation)
Pain score average (VAS), FLACC score average, Comfort B score average (by day and overall for discharge or max 7-day)
1st Successful Physical Therapy Visit (postoperative day #), Signs of Poor Physical Therapy Performance

Magnesium Data
Pre-op Mg Level (most recent), Intra-op Mg Level (average), Intra-op Mg Level (peak)
PICU Magnesium Average Level, Magnesium Peak Level (by day and overall for discharge or max 7-day)
Time Study MgSO4 Bolus Initiated, Dose Study MgSO4 Bolus
Time Study MgSO4 Infusion Initiated, Dose Study MgSO4 Infusion, Time Study MgSO4 Infusion Discontinued
Number of Dose Changes to Study MgSO4 Infusion (and time)
Number of Pauses to Study MgSO4 Infusion to check adverse event
Total Study MgSO4 Dose Received (mg/kg)

Magnesium Side Effects
Peak Respiratory Requirement (by day), Episodes of Respiratory Decline Related to Sedation
Extubation Timing (postoperative day #), Re-intubations (yes/no and #)
Hypotension Presence (yes/no), Number of Vasopressors Required, Vasoactive-Inotropic Score (by day)
Crystalloid Bolus, Colloid Bolus given, Blood Product Bolus given (by day and overall for discharge or max 7-day)
Ionized Calcium, Potassium, Phosphorus average levels (by day and overall for discharge or max 7-day)
Calcium Chloride, Potassium Chloride, Phosphorus, [non-study] MgSO4 Boluses (by day and/or overall for discharge or max 7-day)
Interventions Required for Hyperkalemia (yes/no)
Preoperative Creatinine Level, Creatinine Level 1.5x Upper Limit Normal for Age
Creatinine Average Level (by day and overall for discharge or max 7-day)
Continuous Renal Replacement Therapy Required (yes/no)

Adverse Event
Documented per FDA - Common Terminology Criteria for Adverse Events in review of Progress Notes

*Data collected postoperative days 0 – 7, days considered 7 am – 7 am based on nursing documentation practice at this institution.
