# Supplementary material for: Prolonged magnesium sulfate infusion as adjuvant analgesia in postoperative transplant patients in the pediatric ICU: Preliminary results of a feasibility study
Source: Paediatr Neonatal Pain. 2024 Aug 13;6(4):203–12. doi: 10.1002/pne2.12131 (PMC11645970; doi:10.1002/pne2.12131)
Supplement: Supplementary file 2 — Data S1: [file PNE2-6-203-s001.docx]

**SUPPLEMENTARY MATERIAL**

**Prolonged Magnesium Sulfate Infusion as Adjuvant Analgesia in Post-operative Transplant Patients in the Pediatric ICU: A Feasibility Study**

Table of Contents

Page 2 ……. Supplementary Table 1

Page 3 ……. Supplementary Table 2

Page 4-5 ……. Supplementary Figure 1 (Appendix)

|  | **Hypotensive Episodes** | | **Phenylephrine Bolus (mcg/kg)** | | **Ephedrine Bolus (mcg/kg)** | | **Epinephrine Bolus (mcg/kg)** | | **CaCl Bolus**  **(# interventions)** | | **Crystalloid/Colloid**  **(mL/kg)** | | **Blood Product**  **(#bolus or ml/kg)*** | | **OR KCl Bolus**  **(# interventions)** | | **OR Mg Level**  **(mg/dL)** | | **OR iCa Level**  **(mg/dL)** | | **OR K Level**  **(mmol/L)** | |
| --- | --- | --- | --- | --- | --- | --- | --- | --- | --- | --- | --- | --- | --- | --- | --- | --- | --- | --- | --- | --- | --- | --- |
|  | Mean (SD) | Median | Mean (SD) | Median | Mean (SD) | Median | Mean (SD) | Median | Mean (SD) | Median | Mean (SD) | Median | Mean (SD) | Median | Mean (SD) | Median | Mean (SD) | Peak Range | Mean (SD) | Mean  Range | Mean (SD) | Mean  Range |
| ***TPIAT*** |  |  |  |  |  |  |  |  |  |  |  |  |  |  |  |  |  |  |  |  |  |  |
| MgSO4 | 4.33 (2.55) | 4 | 15.73 (.63) | 11.84 | 0.24 (0.38) | 0.17 | 0 | 0 | 0.56 (0.73) | 0 | 82.05 (32.07) | 69.87 | 0.33 (0.71) | 0 | 0  (0) | 0 | 3.29  (0.15) | 3.3 – 4.2 | 4.41  (0.12) | 4.27 – 4.63 | 3.78  (0.19) | 3.43 – 4.05 |
| Control | 5.21 (2.66) | 5.5 | 13.25 (13.47) | 10.41 | 0.21 (0.4) | 0 | 0.19 (0.15) | 0 | 1.29 (1.24) | 1 | 85.1 (30.42) | 78.37 | 0.41 (0.7) | 0 | 0.21  (0.48) | 0 | 2 | 2 | 4.54  (0.17) | 4.1 – 4.8 | 3.53  (0.22) | 3.1 – 3.9 |
| ***LT*** |  |  |  |  |  |  |  |  |  |  |  |  |  |  |  |  |  |  |  |  |  |  |
| MgSO4 | 2.5 (0.71) | 2.5 | 5.92 (4.1) | 5.92 | 0.076 (0.11) | 0.076 | 0.90 (0.44) | 0.90 | 1 (1.41) | 1 | 17.43 (2.22) | 17.43 | 15.17 (7.32) | 15.17 | 0  (0) | 0 | 3.38  (0.11) | 3.3 – 3.5 | 4.51  (0.01) | 4.5 – 4.51 | 3.86  (0.02) | 3.84 – 3.87 |
| Control | 4.76 (3.21) | 4 | 4.57 (5.77) | 4.32 | 0.026 (0.08) | 0 | 0.41 (0.91) | 0 | 3.57 (2.16) | 3 | 56.77 (33.29) | 55.78 | 65.58 (53.87) | 46.84 | 0.14  (0.36) | 0 | 1.87  (0.19) | 2 – 2.1 | 4.71  (0.34) | 4.16 – 5.46 | 3.74  (0.52) | 2.75 – 4.92 |

***Supplementary Table 1.*** ***Operating room hemodynamic, volume requirement and electrolyte variables***. *Blood product for TPIAT patients in number of bolus interventions vs LT patients in ml/kg based on clinical practice and reporting. CaCl = Calcium Chloride. KCl = potassium chloride. LT = liver transplant. MgSO4 = magnesium sulfate. TPIAT = Total pancreatectomy & islet cell autotransplantation.

|  | **POD0** | | | | **POD1** | | | | **POD2** | | | | **POD3** | | | | **PICU Total** | | | |
| --- | --- | --- | --- | --- | --- | --- | --- | --- | --- | --- | --- | --- | --- | --- | --- | --- | --- | --- | --- | --- |
|  | ***TPIAT*** | | ***LT*** | | ***TPIAT*** | | ***LT*** | | ***TPIAT*** | | ***LT*** | | ***TPIAT*** | | ***LT*** | | ***TPIAT*** | | ***LT*** | |
|  | **MgSO4** | **Control** | **MgSO4** | **Control** | **MgSO4** | **Control** | **MgSO4** | **Control** | **MgSO4** | **Control** | **MgSO4** | **Control** | **MgSO4** | **Control** | **MgSO4** | **Control** | **MgSO4** | **Control** | **MgSO4** | **Control** |
| **Mg peak level**  **(mg/dL)** | 3.06  (0.54) | 1.8  (0.36) | 3.4  (0.57) | 2.01  (0.29) | 2.66  (0.59) | 1.64  (0.28) | 3.2  (0.14) | 2.15  (0.21) | 1.96  (0.42) | 1.7  (0.22) | 3.05  (0.78) | 2.12  (0.3) | 1.68  (0.28) | 1.86  (0.18) | 2.05  (0.21) | 1.96  (0.39) | 2.14  (0.46) | 1.84  (0.15) | 2.64  (0.82) | 1.92  (0.17) |
| **Mg ave. level (mg/dL)** | 2.85  (0.41) | 1.75  (0.33) | 3.25  (0.35) | 1.84  (0.15) | 2.37  (0.46) | 1.57  (0.21) | 2.98  (0.14) | 2.04  (0.22) | 1.89  (0.29) | 1.61  (0.11) | 2.8  (0.49) | 2.07  (0.3) | 1.68  (0.28) | 1.83  (0.18) | 1.95  (0.35) | 1.89  (0.36) | 2.31  (0.37) | 1.83  (0.19) | 2.62  (0.2) | 1.88  (0.17) |
| **iCa ave. level**  **(mg/dL)** | 4.46  (0.31) | 4.55  (0.18) | 4.64  (0.41) | 4.7  (0.3) | 4.54  (0.11) | 4.51  (0.27) | 4.6  (0) | 4.6  (0.34) | 4.78  (0.32) | 4.74  (0.18) | 4.5  (0.14) | 4.36  (0.68) | 4.9  (x) | 4.75  (0.19) | 4.5  (0.28) | 4.7  (0.15) | 4.58  (0.19) | 4.6  (0.18) | 4.61  (0.06) | 4.61  (0.29) |
| **K ave level**  **(mmol/L)** | 3.96  (0.4) | 3.82  (0.4) | 3.6  (0.28) | 3.76  (0.41) | 3.67  (0.23) | 3.47  (0.29) | 3.71  (0.08) | 3.92  (0.44) | 3.59  (0.21) | 3.48  (0.25) | 3.8  (0.14) | 3.62  (0.4) | 3.73  (0.6) | 3.53  (0.35) | 3.68  (0.18) | 3.55  (0.56) | 3.75  (0.11) | 3.6  (0.24) | 3.71  (0.09) | 3.76  (0.35) |
| **Ph ave. level**  **(mg/dL)** | 2.6  (-) | 3.73  (0.49) | 4.43  (0.25) | 4.72  (1.37) | 1.6  (-) | 2.53  (0.69) | 4.53  (0.75) | 4.48  (1.68) | 3.6  (0.57) | 3.08  (0.77) | 5.08  (0.25) | 3.49  (1.41) | 3.55  (0.07) | 3.18  (1.03) | 4.6  (1.7) | 2.99  (0.78) | 3.83  (0.98) | 3.46  (0.77) | 4.59  (0.46) | 3.75  (0.87) |
| **Cr ave. level**  **(mg/dL)** | 0.63  (0.17) | 0.52  (0.15) | 0.49  (0.2) | 0.56  (0.29) | 0.56  (0.11) | 0.5  (0.14) | 0.43  (0.2) | 0.56  (0.41) | 0.52  (0.09) | 0.46  (0.12) | 0.48  (0.25) | 0.6  (0.53) | 0.51  (0.1) | 0.45  (0.12) | 0.67  (0.57) | 0.55  (0.52) | 0.54  (0.11) | 0.48  (0.13) | 0.51  (0.31) | 0.57  (0.4) |
| **CaCl Boluses** | 0.22  (0.67) | 0.088  (0.29) | 0.5  (0.71) | 0.476  (1.12) | 0.22  (0.44) | 0.18  (0.58) | 0  (0) | 0.42  (0.87) | 0  (0) | 0.029  (0.03) | 0  (0) | 0.095  (0.3) | 0  (0) | 0  (0) | 0  (0) | 0.05  (0.22) | 0.33  (0.71) | 0.29  (0.84) | 0  (0) | 1.24  (2.41) |
| **Mg Boluses (non-protocol)** | --- | --- | --- | --- | --- | --- | --- | --- | --- | --- | --- | --- | --- | --- | --- | --- | 0.33  (0.5) | 0.71  (1.55) | 1  (1.41) | 2.86  (2.99) |
| **KCl Boluses** | --- | --- | --- | --- | --- | --- | --- | --- | --- | --- | --- | --- | --- | --- | --- | --- | 1  (0.71) | 2.79  (0.84) | 1  (1.41) | 4.9  (6.6) |
| **Ph Boluses** | --- | --- | --- | --- | --- | --- | --- | --- | --- | --- | --- | --- | --- | --- | --- | --- | 0.22  (0.67) | 0.5  (2) | 1  (1.41) | 3.62  (5.35) |

***Supplementary Table 2. Postoperative lab data and electrolyte supplementation***. Values presented as Average (Standard Deviation). Phosphorus boluses include both sodium-Ph and potassium-Ph boluses. Mg, K, and Ph boluses were collected only as cumulative PICU tallies, not individually by day. TPIAT = total pancreatectomy & islet cell autotransplantation. Ave. = average. CaCl = calcium chloride. Cr = creatinine. iCa = ionized calcium. K = potassium. KCl = potassium chloride. LT = Liver transplantation. Mg = magnesium. MgSO4 = magnesium sulfate. Ph = phosphorus. POD = postoperative day #.

***Appendix 1. List of Study Variables***

**Demographics**

Gender

Age, Weight, Height

Date of Surgery

Transplant received (TPIAT/Liver)

Abdomen Left Open after Surgery (y/n)

PICU Length of Stay

**OR Data**

Ionized Calcium, Potassium Levels (average)

Total Fluid [non-blood] Required (mL)

Total Blood Product Required (mL)

Number of Pressor Infusions Required, Peak Pressor Doses

Vasoactive-Inotropic Score

Ephedrine, Phenylephrine, Vasopressin, Epinephrine, and Norepinephrine Bolus Rescue Doses

Bradycardia Present (yes/no), Estimated Time Bradycardic (minutes, % total)

Sustained Hypotensive events

Total OR time (minutes)

Calcium Chloride, Potassium Chloride Boluses (# received)

**Opioid/Sedation Consumption and Side Effect Data**

Opioid Class (by day and overall)

PO Morphine Equivalent/kg (by day, for postoperative day 0 – 2, and overall for discharge or max 7-day)

% opioid scheduled vs PRN/demand

PCA use (yes/no), PCA deliveries vs demands

Ketamine Dosage (mg/kg by day and overall for discharge or max 7-day), Ketamine Infusion Use (yes/no)

Dexmedetomidine Peak dose (mg/kg/hr, by day), Dexmedetomidine Total Dose (mg/kg, overall for discharge or max 7-day)

Paravertebral Block Use (yes/no), Number of paravertebral Block Interventions (considered increase in infusion dose or boluses)

Number of Stools (by day and overall for discharge or max 7-day), Day of 1^st^ Stool (Postoperative day #)

Suppository or Enemas used (by day and overall for discharge or max 7-day)

Ileus Documented (yes/no)

Initiation of Enteral Feeds (postoperative day #), Reached Goal Feeds (postoperative day # or did-not-reach)

Emeses (by day and overall for discharge or max 7-day)

Ondansetron or Granisetron doses, Prochlorperazine or Metoclopramide doses (by day and overall for discharge or max 7-day)

Scopalamine patch use (yes/no, by day and overall for discharge or max 7-day)

Cyproheptadine doses, Diphenhydramine doses (by day and overall for discharge or max 7-day)

Benzodiazepine doses (by day and overall for discharge or max 7-day), Midazolam Infusion use (yes/no)

Acetaminophen doses (by day and overall for discharge or max 7-day)

Ketorolac doses, Ibuprofen doses, Celecoxib doses (by day and overall for discharge or max 7-day)

Gabapentinoid doses (by day and overall for discharge or max 7-day)

Tricyclic antidepressant doses (by day and overall for discharge or max 7-day)

Naloxone used (yes/no)

Urinary Retention (yes/no)

Delirium (yes/no per progress note documentation)

Pain score average (VAS), FLACC score average, Comfort B score average (by day and overall for discharge or max 7-day)

1^st^ Successful Physical Therapy Visit (postoperative day #), Signs of Poor Physical Therapy Performance

**Magnesium Data**

Pre-op Mg Level (most recent), Intra-op Mg Level (average), Intra-op Mg Level (peak)

PICU Magnesium Average Level, Magnesium Peak Level (by day and overall for discharge or max 7-day)

Time Study MgSO4 Bolus Initiated, Dose Study MgSO4 Bolus

Time Study MgSO4 Infusion Initiated, Dose Study MgSO4 Infusion, Time Study MgSO4 Infusion Discontinued

Number of Dose Changes to Study MgSO4 Infusion (and time)

Number of Pauses to Study MgSO4 Infusion to check adverse event

Total Study MgSO4 Dose Received (mg/kg)

**Magnesium Side Effects**

Peak Respiratory Requirement (by day), Episodes of Respiratory Decline Related to Sedation

Extubation Timing (postoperative day #), Re-intubations (yes/no and #)

Hypotension Presence (yes/no), Number of Vasopressors Required, Vasoactive-Inotropic Score (by day)

Crystalloid Bolus, Colloid Bolus given, Blood Product Bolus given (by day and overall for discharge or max 7-day)

Ionized Calcium, Potassium, Phosphorus average levels (by day and overall for discharge or max 7-day)

Calcium Chloride, Potassium Chloride, Phosphorus, [non-study] MgSO4 Boluses (by day and/or overall for discharge or max 7-day)

Interventions Required for Hyperkalemia (yes/no)

Preoperative Creatinine Level, Creatinine Level 1.5x Upper Limit Normal for Age

Creatinine Average Level (by day and overall for discharge or max 7-day)

Continuous Renal Replacement Therapy Required (yes/no)

**Adverse Event**

Documented per FDA - Common Terminology Criteria for Adverse Events in review of Progress Notes

*Data collected postoperative days 0 – 7, days considered 7 am – 7 am based on nursing documentation practice at this institution.
